# Isolation and functional characterization of a high affinity urea transporter from roots of *Zea mays*

**DOI:** 10.1186/s12870-014-0222-6

**Published:** 2014-08-29

**Authors:** Laura Zanin, Nicola Tomasi, Corina Wirdnam, Stefan Meier, Nataliya Y Komarova, Tanja Mimmo, Stefano Cesco, Doris Rentsch, Roberto Pinton

**Affiliations:** Dipartimento di Scienze Agrarie e Ambientali, University of Udine, via delle Scienze 208, I-33100 Udine, Italy; Institute of Plant Sciences, University of Bern, Altenbergrain 21, CH-3013 Bern, Switzerland; Faculty of Science and Technology, Free University of Bolzano, Piazza Università 5, I-39100 Bolzano, Italy

**Keywords:** Corn, High affinity transport system, DUR3, Maize, Nitrogen (N), Root, Urea

## Abstract

**Background:**

Despite its extensive use as a nitrogen fertilizer, the role of urea as a directly accessible nitrogen source for crop plants is still poorly understood. So far, the physiological and molecular aspects of urea acquisition have been investigated only in few plant species highlighting the importance of a high-affinity transport system. With respect to maize, a worldwide-cultivated crop requiring high amounts of nitrogen fertilizer, the mechanisms involved in the transport of urea have not yet been identified. The aim of the present work was to characterize the high-affinity urea transport system in maize roots and to identify the high affinity urea transporter.

**Results:**

Kinetic characterization of urea uptake (<300 μM) demonstrated the presence in maize roots of a high-affinity and saturable transport system; this system is inducible by urea itself showing higher Vmax and Km upon induction. At molecular level, the ORF sequence coding for the urea transporter, *ZmDUR3*, was isolated and functionally characterized using different heterologous systems: a *dur3* yeast mutant strain, tobacco protoplasts and a *dur3 Arabidopsis* mutant. The expression of the isolated sequence, *ZmDUR3*-ORF, in *dur3* yeast mutant demonstrated the ability of the encoded protein to mediate urea uptake into cells. The subcellular targeting of DUR3/GFP fusion proteins in tobacco protoplasts gave results comparable to the localization of the orthologous transporters of *Arabidopsis* and rice, suggesting a partial localization at the plasma membrane. Moreover, the overexpression of *ZmDUR3* in the *atdur3-3 Arabidopsis* mutant showed to complement the phenotype, since different *ZmDUR3*-overexpressing lines showed either comparable or enhanced ^15^[N]-urea influx than wild-type plants. These data provide a clear evidence *in planta* for a role of ZmDUR3 in urea acquisition from an extra-radical solution.

**Conclusions:**

This work highlights the capability of maize plants to take up urea via an inducible and high-affinity transport system. ZmDUR3 is a high-affinity urea transporter mediating the uptake of this molecule into roots. Data may provide a key to better understand the mechanisms involved in urea acquisition and contribute to deepen the knowledge on the overall nitrogen-use efficiency in crop plants.

**Electronic supplementary material:**

The online version of this article (doi:10.1186/s12870-014-0222-6) contains supplementary material, which is available to authorized users.

## Background

By 2050, the global population is expected to be 50% higher than at present and global grain demand is projected to double (http://www.fao.org/fileadmin/templates/wsfs/docs/Issues_papers/HLEF2050_Global_Agriculture.pdf).

Today the productivity of crops is based on the application of high amounts of industrially produced nitrogen (N) fertilizer, even though crop plants utilize only 30-40% of the applied N [1]. As a consequence, the wide use of synthetic N fertilizer has led to negative impacts on the environment and on farmer economies. In addition, the N use efficiency (NUE) of cereal crops has declined in the last 50 years [2].

Based on these considerations, crop yield needs to be improved in a more cost-effective and eco-compatible way. This goal could be achieved by increasing the NUE of cereals and optimizing the acquisition of naturally occurring and applied N. Reducing the amount of fertilizers in maize culture will have economic and environmental benefits. In particular combining reduced fertilizer application and breeding plants with better NUE is one of the main goals of research in plant nutrition [3].

Urea is the most frequently used N fertilizer in the world, with annual amounts of over 50 million tons accounting for more than 50% of the world N-fertilizer consumption (www.fertilizer.org/Statistics). The great increase in urea-fertilizer use during the last decades is mainly due to its competitive price and the high N content (46% of mass), that allow reducing transport and distribution costs [4]. Besides the chemical input as fertilizer, urea is also a natural organic molecule synthesized by most organisms [5,6]. In plants, urea represents an important metabolic intermediate produced during N-recycling [6], while in mammals the urea production is associated with the detoxification of N compounds [7].

Although urea might be derived from both natural and chemical syntheses, in the soil it usually occurs only at micromolar concentrations (less than 10 μM [8-10]). Also in soils of fertilized crop-plants, the urea concentration is maintained at low levels (up to 70 μM [11]). In part, this is due to the presence of microbial ureases in the soil solution, which rapidly hydrolyse urea into carbon dioxide and ammonia. However, low concentrations of urea could remain in soils also after enzymatic degradation, since the microbial urease activity shows an affinity constant in the millimolar range [12]. As evolutionary adaptation, plants might have developed strategies to use this diluted but available N source through high affinity urea uptake systems [5].

Only few studies have investigated the molecular basis of urea transporters in higher plants. The first research was published by Liu *et al.* [13] reporting the cloning and characterization of a high affinity urea transporter of *Arabidopsis*, called *AtDUR3*. The coding sequence of *AtDUR3* showed weak homology to an ortholog of *Saccharomyces cerevisiae* (*ScDUR3*), a member of the sodium-solute symporter (SSS) gene family, which is widespread in microorganisms, animals, and humans [14,15]. Members of the SSS family have been described to transport a wide range of solutes, such as sugars, amino acids, nucleosides, inositols, vitamins, anions, and urea [14,16,17]. AtDUR3 showed no significant homology to any other protein of *Arabidopsis* [13]. Similarly, in the rice genome, *OsDUR3* is the only gene that has significant homology to *AtDUR3*, suggesting that plant DUR3 proteins might represent a transporter subfamily consisting of only one member [18,19]. To date, in higher plants only *Arabidopsis* and rice DUR3 have been characterized at the molecular and physiological level [13,18,19].

The aim of the present work was to identify and functionally characterize the high affinity transport system involved in urea acquisition in maize. To do this, the kinetic properties of urea uptake in intact maize roots were determined. The putative urea transporter *ZmDUR3*-ORF was isolated and its localization analysed using GFP-fusion proteins; its capability to transport urea was demonstrated by expression in heterologous systems, *i.e. dur3 Saccharomyces cerevisiae* and *Arabidopsis thaliana* mutants.

## Results

### Urea acquisition in maize plants

**Figure 1:**
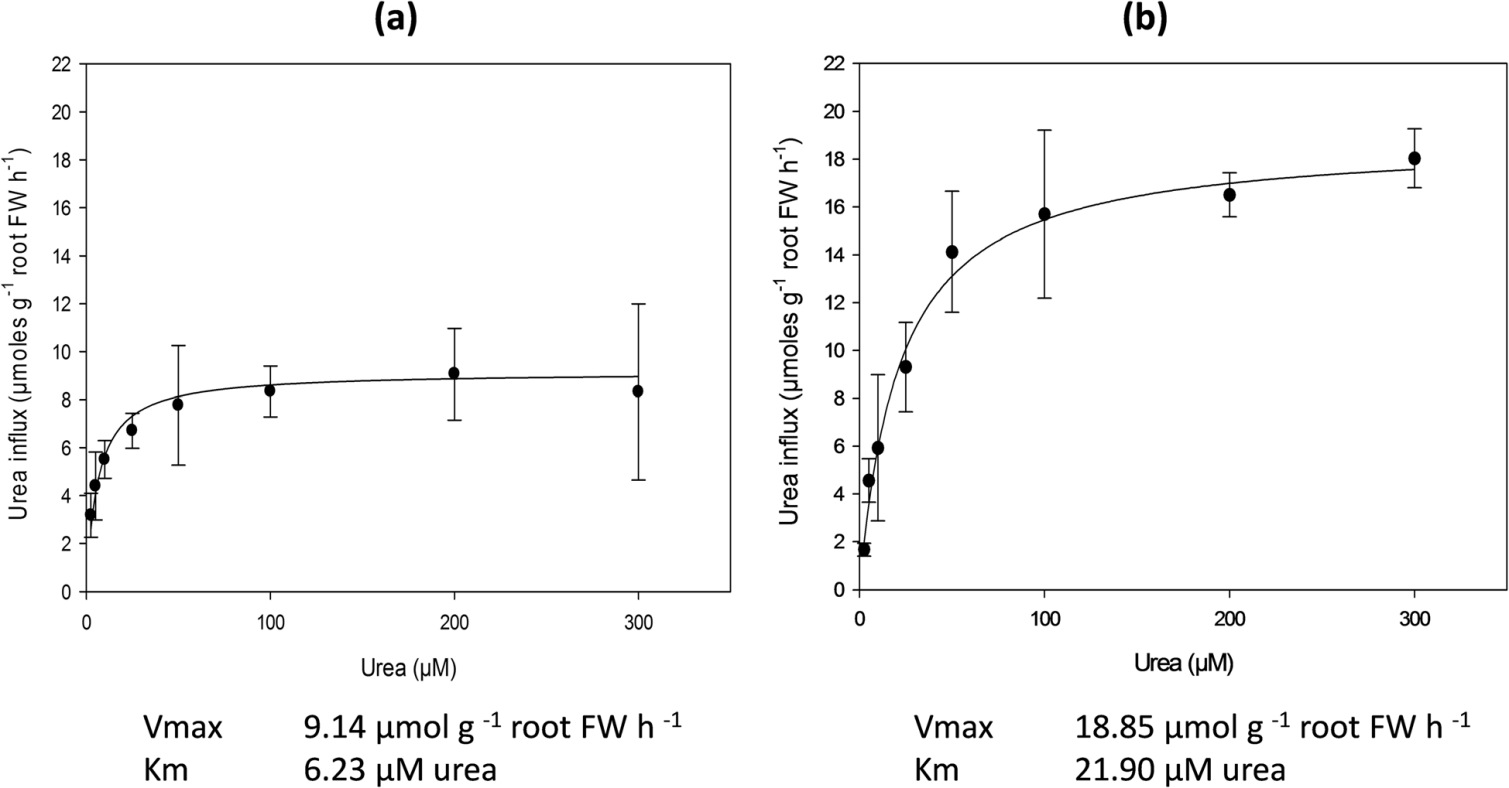
**Kinetic assay of urea uptake by maize roots.** The concentration-dependent uptake was measured using 5-day-old maize plants exposed for 4 h to a nutrient solution supplied with 1 mM urea as a sole nitrogen source **(b)** or not (control plants, **a)**. Subsequently roots were incubated for 10 minutes in the assay solution containing urea at different concentrations (2.5-5-10-25-50-100-200-300 μM). Values are means ± SD (*n* = 3).

To evaluate the capacity of maize roots to take up urea, a concentration dependent net-influx analysis was performed using 5-day-old plants grown in N-free nutrient solution. Before the uptake experiment, plants were exposed for 4 hours to a nutrient solution containing 1 mM urea as sole N source (urea treatment), or without N (control). Net uptake rates were determined measuring urea depletion from assay solutions, containing 2.5 to 300 μM urea (Figure 1).

In roots of control plants, the uptake rates of urea showed a typical saturation kinetic corresponding to the Michaelis-Menten model (Figure 1a). Interestingly, the exposure of roots to 1 mM urea before the uptake assay modified the kinetic parameters (Figure 1b). Indeed the net urea influx in roots of urea pre-treated plants was more than 2 fold higher compared to that measured in control plants, with Vmax values of about 19 and 9 μmol urea g^−1^ fresh weight (FW) h^−1^, respectively. The urea pre-treatment also affected the affinity, which decreased in pre-treated plants more than 3.5 times with respect to control plants (Km about 22 μM and 6 μM, respectively).

In order to independently verify the capacity of maize plants to acquire urea, ^15^[N] -labelled urea was supplied in the nutrient solution. After 24 hours of treatment the accumulation of ^15^ N was 327.3 (±13.8) mg 100 g^−1^ dry weight (DW) in shoots and 421.1 (±18.4) mg 100 g^−1^ DW in roots. During the time span of the experiment, no detectable degradation of urea occurred in the nutrient solution (data not shown). In this way considering ^15^ N-data, maize plants took up around 25 μmol 100 mg^−1^ DW of urea from the external solution.

To investigate the contribution of urea taken up by roots in terms of intact molecule in the plants, the concentration of urea in roots and shoots of maize plants was analysed (Additional file 1: Figure S1). After 24 hours comparable amounts of urea were detected in urea- and control- treated plants. Nevertheless, the concentrations of urea within maize tissues, roots or shoots, were significantly different during the time span of the experiment. After 4 and 8 hours, the urea concentration decreased in roots and increased in shoots of urea-treated plants. This modulation in urea content might suggest a translocation of urea (as intact molecule) even if a higher degradation in roots and a synthesis in shoots cannot be excluded.

### *In silico* identification of a maize urea transporter

**Figure 2:**
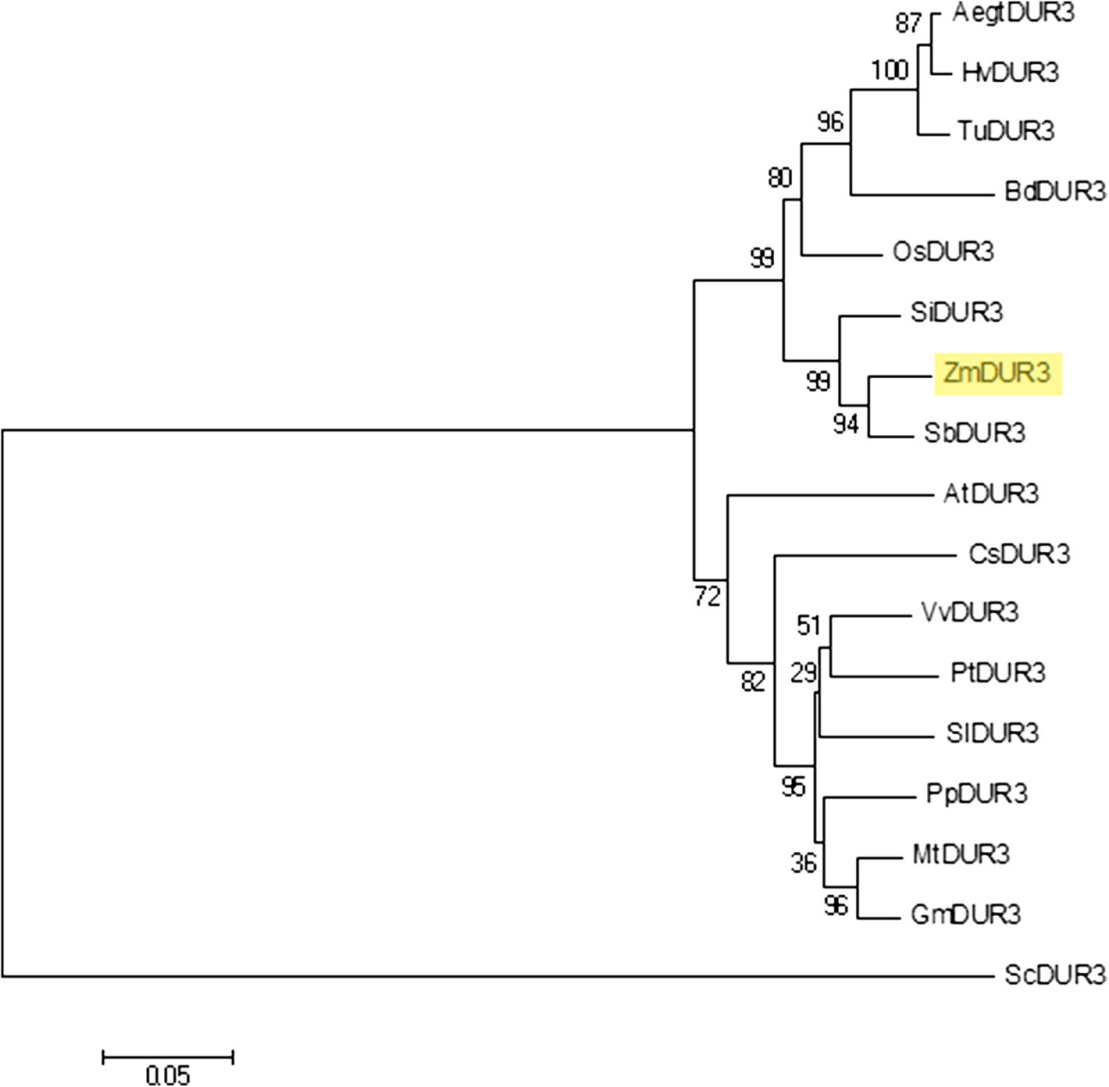
**Phylogenetic tree of DUR3 urea transporters.** A phylogentic analyses was performed using the DUR3 amino acid sequences of *Saccharomyces cerevisiae* (Sc, AAA34582), *Zea mays* (Zm, KJ652242), *Oryza sativa* (Os, NP_001065513), *Arabidopsis thaliana* (At, NP_199351) and putative DUR3 orthologs from *Aegilops tauschii* (Aegt, EMT22254), *Triticum urartu* (Tu, EMS63712.1), *Hordeum vulgare* (Hv, BAJ94433.1), *Brachypodium distachyon* (Bd, XP_003571687), *Setaria italica* (Si, XP_004965066), *Sorghum bicolor* (Sb, XP_002438118), *Cucumis sativus* (Cs, XP_004146194.1), *Vitis vinifera* (Vv, XP_002263043), *Populus trichocarpa* (Pt, XP 002303472.1), *Solanum lycopersicum* (Sl, XP_004245999), *Prunus persica* (Pp, EMJ11521.1), *Medicago truncatula* (Mt, XP_003612583), *Glycine max* (Gm, XP_003523904). The tree was constructed by aligning the protein sequences by Clustal-W and the evolutionary history was inferred using the Neighbor-Joining method. The percentage of replicate trees in which the associated taxa clustered together in the bootstrap test (1000 replicates) are shown next to the branches. The tree is drawn to scale, with branch lengths in the same units as those of the evolutionary distances used to infer the phylogenetic tree. The evolutionary distances were computed using the Poisson correction method and are in the units of the number of amino acid substitutions per site.

With the aim to identify a high affinity urea transporter from maize, an *in-silico* search was performed based on sequence similarity with *AtDUR3* (At5g45380) using the BLAST algorithm on the Aramemnon plant membrane protein database (http://aramemnon.botanik.uni-koeln.de/index.ep, ARAMEMNON v. 7.0© [20]). In the maize genome, only one predicted sequence coding for a DUR3 homolog (putative transcript AC202439.3_FGT006) was identified on chromosome 6 (113,848,061-113,853,627). The expression of *ZmDUR3* was confirmed by several EST-sequences present in the Nucleotide EST Database from GenBank (dbEST, http://www.ncbi.nlm.nih.gov/nucest): BQ164112, BQ164020, FL011289, FL448872, DV550376, AW400387, BQ163839, BQ163822 and FL011290. Most ESTs covered the 3′-region of AC202439.3_FGT006 while only FL011289 and FL011290 aligned at the 5′-region. We thus referred to this gene as *ZmDUR3* (Figure 2). When widening the search only a single predicted DUR3 ORF was found within each of the plant species analysed. The phylogenetic analysis revealed that putative DUR3 proteins are closely related among monocots, such as maize, rice, wheat, barley and millet (Figure 2), with more than 80% identity at the amino acid level.

### Expression pattern of *ZmDUR3* in maize tissues

**Figure 3:**
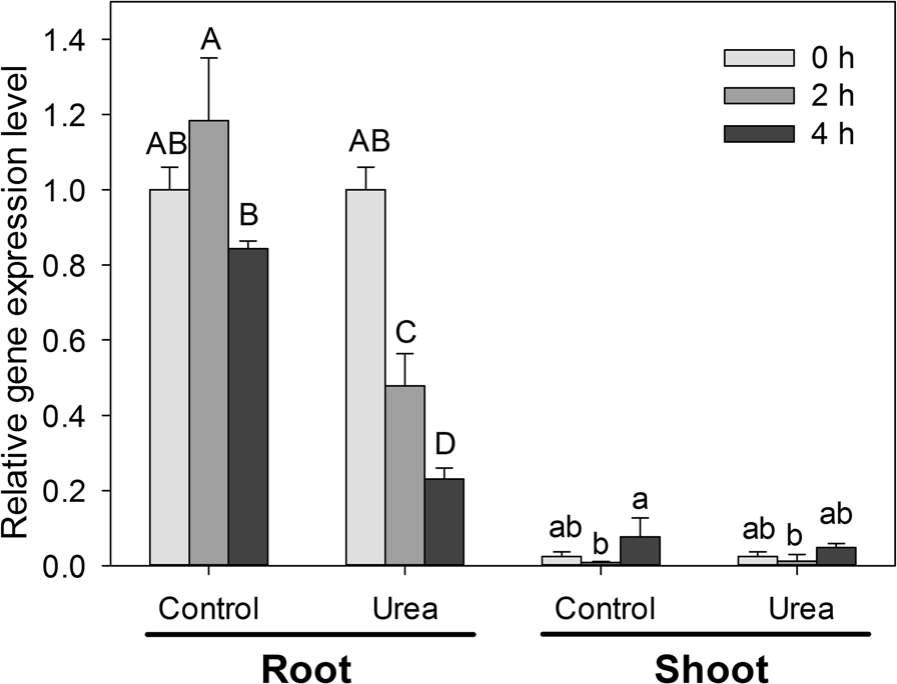
**Transcriptional analyses of** ***ZmDUR3*** **in root and shoots of maize in response to urea treatment.** 5 day-old plants were exposed for a maximum of 4 hours to nutrient solution without addition of any N source (*Control* plants) or supplied with 1 mM urea (*Urea* treated plants). Gene mRNA levels were normalized with respect to the mean transcript level of the housekeeping gene *ZmRPS4*; relative changes in gene transcript levels were calculated on the basis of the mean transcript level of *ZmRPS4* in roots of *Control* plants at 0 hour (Relative gene expression = 1). Values are means ± SD of three independent experiments (ANOVA, Student-Newman-Keuls*, P < 0.05, n* = 3). *Capital letters* are referred to the statistical differences in the roots, while *lower letters* are referred to shoots.

As reported in Figure 3, real time RT-PCR data show the expression pattern of *ZmDUR3* in maize plants up to 4 hours of root exposure to urea. The highest gene expression level of *ZmDUR3* was reached in roots while in leaves the transcript amount was at least an order of magnitude lower.

Up to 4 hours of urea treatment, the presence of the nitrogen source in the external solution induced a significant down regulation of the gene expression. On the other hand, in urea and control leaves the expression levels were comparable and not significantly influenced by the treatment.

### The coding sequence of *ZmDUR3* was isolated from maize root mRNA

Using gene specific primers, a transcript from maize root was amplified by RT-Assembly-PCR and cloned into the yeast expression vector pDR197 [21]. The sequencing results showed an open reading frame of 2196-bp, *ZmDUR3*-ORF [GenBank: KJ652242], coding for 731 amino acids. The alignment with the genomic sequence (AC202439.3_FG006) revealed four exon regions of 192, 108, 663 and 1233 bp. The length and the location of the exons were different from those predicted (Additional file 2: Figure S2). In addition, in comparison to the predicted cDNA (AC202439.3_FGT006), the isolated *ZmDUR3*-ORF contained three non-synonymous substitutions in the nucleotide sequence, modifying the following amino acids: K149N; A167V; Q559H. The nucleotide responsible for the Q559H modification was also detected in a maize EST sequence (BQ164112). The region containing the other two substitutions was not covered by ESTs. However, the presence of asparagine (N) and histidine (H) instead of lysine (K) and glutamine (Q), respectively, was also found in the amino-acid sequence of rice *OsDUR3* [19].

Blast analysis revealed that the *ZmDUR3* cDNA had a high similarity with *OsDUR3* (84% nucleotide sequence identity with a 94% of query coverage). Similar percentages were also observed at amino acid level with an identity of 83 and 75% to OsDUR3 and AtDUR3, respectively (Additional file 3: Figure S3). ZmDUR3 comprises 731 amino acids containing fifteen predicted transmembrane spanning domains (TMSDs) with outside orientation of the N-terminus (prediction performed by TOPCONS, http://topcons.cbr.su.se/, and confirmed by *TMHMM 2.0,*http://www.cbs.dtu.dk/services/TMHMM/). The comparison between ZmDUR3 and the rice ortholog OsDUR3 (721 amino acids) revealed a similar predicted topology (Additional file 4: Figure S4), especially with respect to the number of TMSDs, and N- and C-terminus orientation.

### Functional characterization of ZmDUR3

The functional characterization was performed using different approaches in heterologous systems: i) functional complementation of a *Saccharomyces cerevisiae dur3* mutant, ii) subcellular localization of ZmDUR3/GFP (Green Fluorescent Protein) fusion proteins in *Nicotiana tabacum* protoplasts and iii) *35sCaMV:: ZmDUR3* overexpression in the *atdur3* mutant line of *Arabidopsis thaliana*.

**Figure 4:**
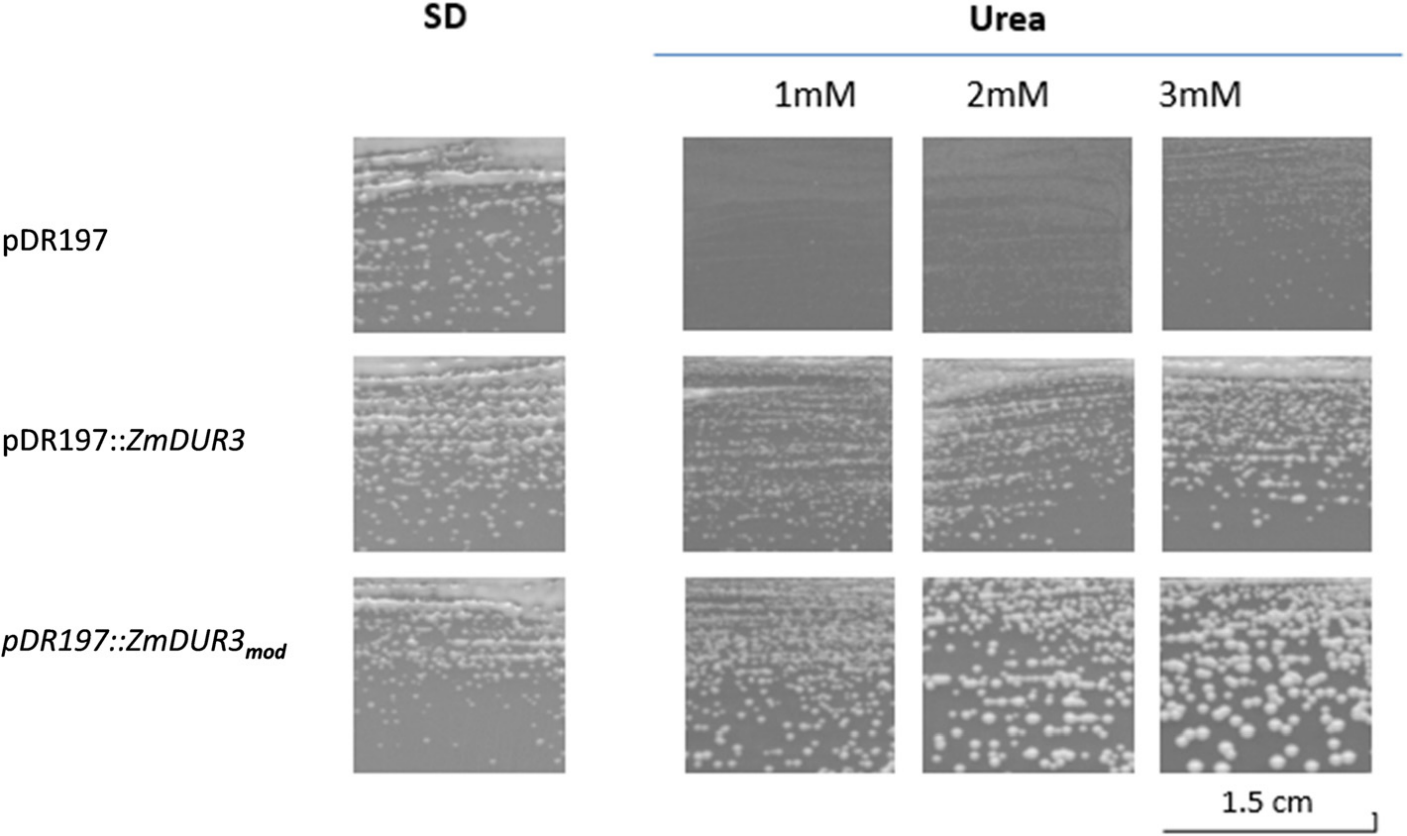
**ZmDUR3 mediates urea uptake in** ***S. cerevisiae*** **.** Growth of the urea uptake-deficient strain YNVW1 expressing *ZmDUR3* and *ZmDUR3*_*mod*_. The mutant YNVW1 transformed with the vector pDR197 (first row), and pDR197 carrying ORFs *ZmDUR3* (middle row) or *ZmDUR3*_*mod*_ (third row). Medium contained 0.5% of ammonium sulphate (SD) or urea at three different concentrations (1, 2 or 3 mM urea) as a sole nitrogen source. Pictures were taken after 5 days of incubation.

In order to verify the ability to transport urea, the *ZmDUR3*-ORF was expressed in a *dur3-*mutant strain of *S. cerevisiae*, as described previously by Liu *et al.* [13]. The mutant YNVWI (Δura3, Δdur3) is defective in urea uptake and cannot grow on less than 5 mM urea as sole N source [13]. Results showed that the *dur3* mutant strain transformed with the vector pDR197 barely grew on a medium containing 1, 2 or 3 mM urea. On the other hand, the heterologous expression of *ZmDUR3-*ORF enabled YNVWI to grow well on urea medium (Figure 4). Moreover, since *ZmDUR3* has a high GC-content (around 80% GC content in the first 100 bp), the level of heterologous expression in other organisms may be limited. So, to reduce the GC content and favour the expression of *ZmDUR3*, 48 nucleotides in the first 216 nt of *ZmDUR3* were modified. These modifications are all synonymous substitutions occurring only at nucleotide level (as specified in the *Methods*). A great improvement in the yeast growth on urea medium was observed transforming YNVWI with a modified version of *ZmDUR3-*ORF (called *ZmDUR3*_*mod*_*-*ORF, Figure 4).

The YNVWI mutant expressing *ZmDUR3-*ORFs (*ZmDUR3*- and *ZmDUR3*_*mod*_-transformants) did not show any apparent growth difference on medium supplemented with 0.5% ammonium sulphate, as N source. When grown on selective plates supplemented with urea as a sole N source, growth differences between *ZmDUR3*- and *ZmDUR3*_*mod*_-transformants became apparent. In particular, the size of the colonies of *ZmDUR3*_*mod*_-transformants was larger in comparison to those of the native *ZmDUR3*-ORF, and this different growth was visible for all urea concentrations tested.

### Transient expression of ZmDUR3/GFP fusion proteins in tobacco protoplasts

**Figure 5:**
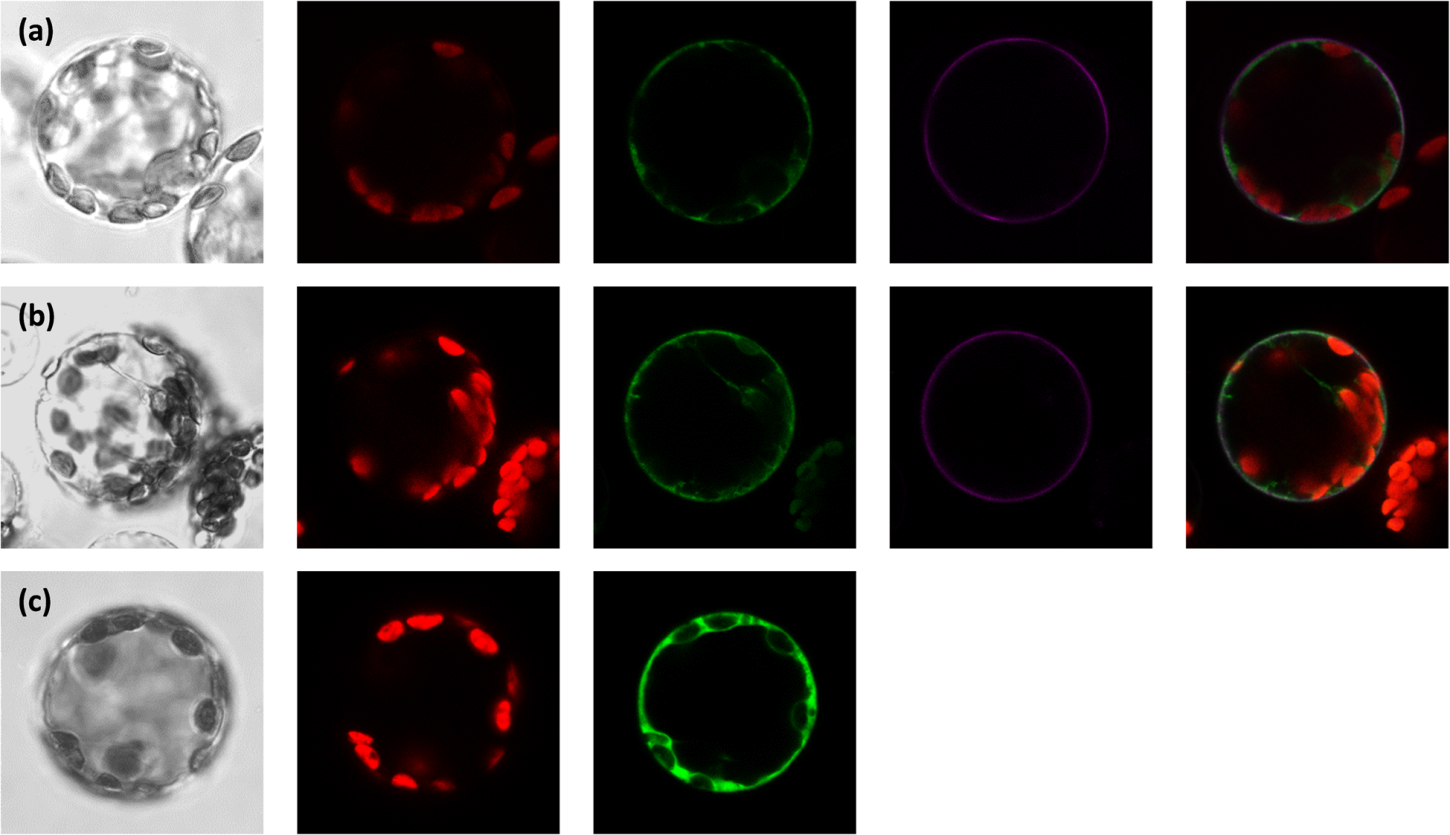
**Localization of ZmDUR3/GFP fusion proteins in tobacco protoplast. (a)** Co-localization of ZmDUR3-GFP and plasma membrane localized AtPTR1-YFP, **(b)** GFP-ZmDUR3 and AtPTR1-YFP, and **(c)** free GFP. Fluorescence was detected using a confocal laser-scanning microscope: bright-field images (first column), chlorophyll fluorescence (red signal, second column), GFP-fluorescence (green signal, third column); YFP-fluorescence (purple signal, as control for plasma membrane localization, fourth column) are shown. In the last column, merged images show chlorophyll fluorescence (red), GFP-fluorescence (green) and YFP-fluorescence (purple). Diameter of protoplasts was approximately 40 μm.

Functional complementation of the yeast mutant YNVWI by ZmDUR3 indicated that at least in a heterologous system the transporter is localized at the plasma membrane. To confirm this subcellular localization, N- and C-terminal fusion proteins of ZmDUR3 and GFP (Green Fluorescent Protein) were transiently expressed in tobacco (*N. tabacum*) protoplasts (Figure 5a,b). Tobacco protoplasts were also transformed with AtPTR1-YFP [22] or with free GFP, which were used as plasma membrane and cytosolic control, respectively (Figure 5).

In free-GFP expressing protoplasts the fluorescent signal was localized in the cytoplasm (Figure 5c). In protoplasts expressing ZmDUR3-GFP (Figure 5a) and GFP-ZmDUR3 (Figure 5b) plasma membrane localization could not be unequivocally demonstrated, since the green fluorescence was mostly confined to internal membranes. The functionality of *ZmDUR3*_*mod*_*/GFP* constructs was verified in *dur3-*yeast mutant.

### Overexpression of *ZmDUR3* in *Arabidopsis* mutant line *atdur3-3*

In order to test the activity of ZmDUR3 *in planta*, *ZmDUR3*_*mod*_ was overexpressed in a *dur3* mutant line of *Arabidopsis*. The *atdur3-3* mutant is defective in the endogenous urea transporter AtDUR3 and showed impaired growth on a medium with urea (<5 mM) as sole N source [18]. In particular the mutant line showed a slow development and chlorotic leaves at 0.5 and 1 mM urea [18], suggesting a condition of N deficiency.

**Figure 6:**
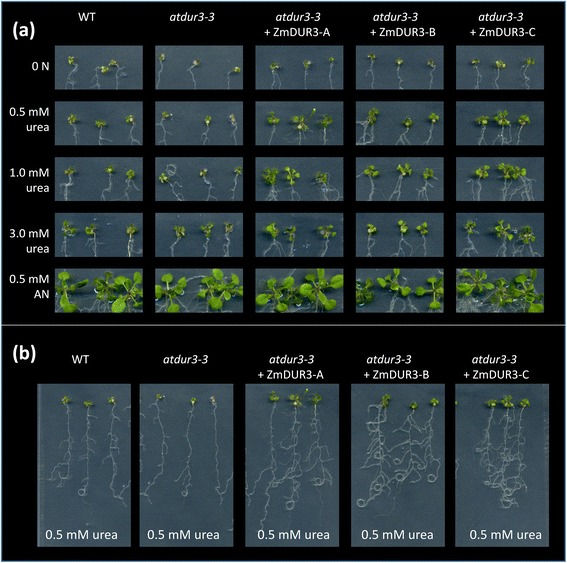
**Growth of** ***ZmDUR3***_***mod***_ **-expressed in the** ***dur3-3 Arabidopsis*** **mutant.** The *Arabidopsis dur3* mutant, *atdur3-3* [18], was transformed with *ZmDUR3*_*mod*_-ORF under the control of the CaMV 35S-promoter. **(a)** Growth of the wild type Col-0 (WT), *atdur3-3* mutant line and three *ZmDUR3*_*mod*_-overexpressing lines (*atdur3-3* + ZmDUR3-A, −B, −C) on sterile half strength MS medium supplied with 1 μM NiCl_2_ and 50 μM NO_3_^−^ and different concentrations of urea or 0.5 mM ammonium nitrate (AN) as a sole N source. **(b)** Effect of urea treatment on root morphology in *Arabidopsis* plants grown with 0.5 mM urea. Plants were grown for 16 days on nutrient agar-medium.

**Figure 7:**
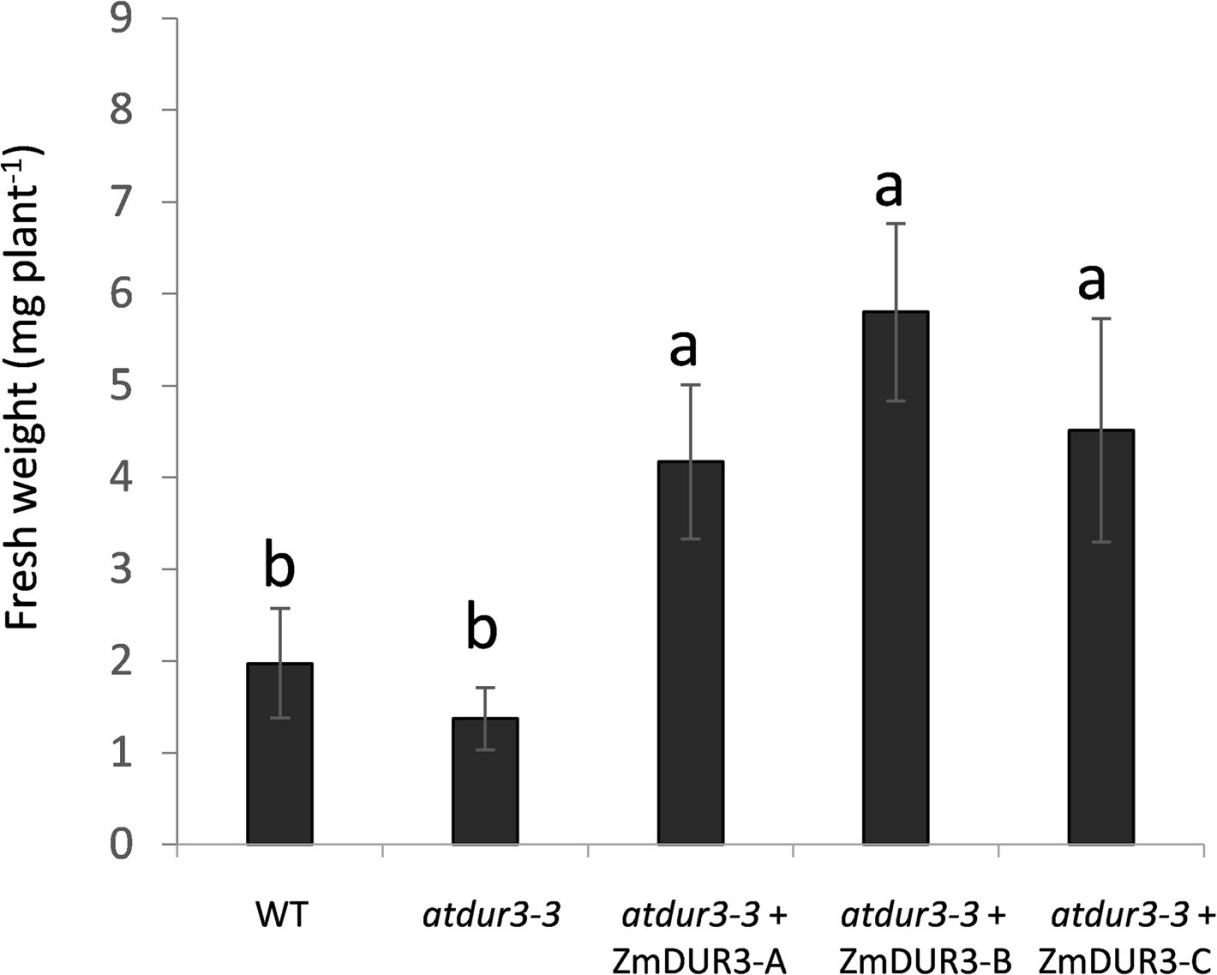
**Effect of urea treatment on biomass production of** ***Arabidopsis*** **plants grown on 0.5 mM urea.** *Arabidopsis* plants were grown on sterile half strength MS medium supplemented with 1 μM NiCl_2_ and 50 μM NO_3_^−^ plus 0.5 mM urea as sole N sources (same growth conditions described for Figure 6b). The fresh weights of 14 plants were measured after 16 days. Data are mean ±SD of three independent experiments and different letters above the bars indicate statistically significant differences (ANOVA, Student-Newman-Keuls*, P < 0.05, n*=3).

Three independent *35sCaMV: ZmDUR3*_*mod*_-overexpressing lines were tested: line-A, line-B and line-C. Plants were grown for 16 days on sterile half strength MS medium without any additional N, or supplemented with urea at three different concentrations (0.5, 1.0 or 3.0 mM urea) or 0.5 mM ammonium nitrate. The complementation assay demonstrated that in all three overexpression lines the capacity to grow on a medium supplemented with 0.5 mM and 1 mM urea was restored (Figure 6a). On agar plates without N supply, all plants showed a poor development of shoots and roots and symptoms of N deficiency appeared. On medium containing 0.5 mM urea, wild type shoots developed slightly better than *dur3* shoots, as previously described by Kojima *et al.* [18]. At 0.5 mM urea, the *ZmDUR3*_*mod*_-overexpressing lines grew better than wild type plants with a good development of shoots and with a higher root proliferation (Figure 6b). It is interesting to note that on agar plates supplemented with 0.5 mM urea, overexpression lines showed a higher biomass production with a significantly higher fresh weights than wild type or *atdur3-3* mutant plants (Figure 7). No detectable differences were observed among all *Arabidopsis* lines tested when plants were grown on 3 mM urea or on 0.5 mM ammonium nitrate (Figure 6a).

**Figure 8:**
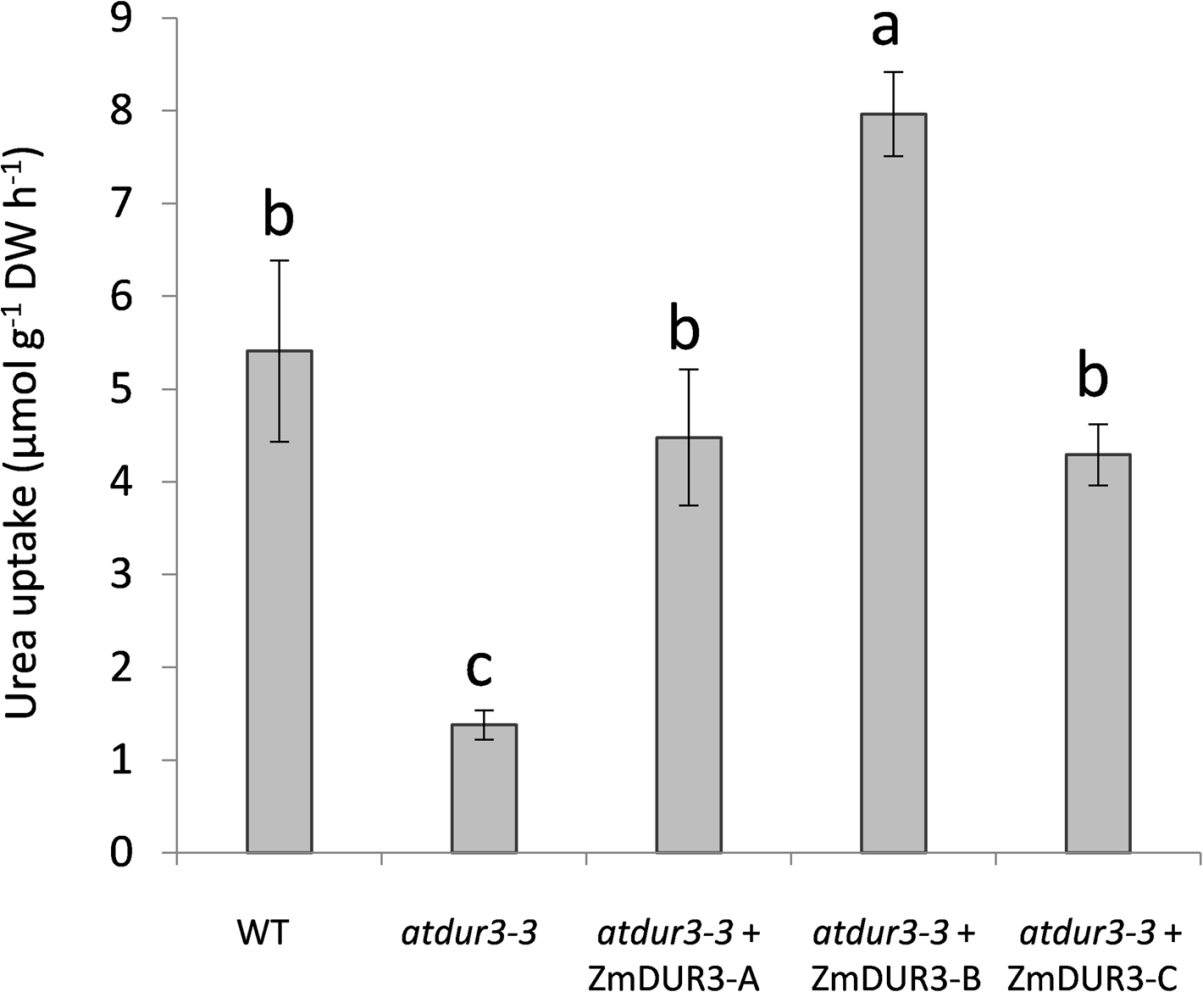
^**15**^ **[N]-urea influx in** ***Arabidopsis*** **plants.** Urea uptake into roots was determined using 6-weeks-old plants of wild type Col-0 (WT), *atdur3-3* mutant line and three *ZmDUR3*_*mod*_-transformed lines (*atdur3-3* + ZmDUR3-A, −B, −C) grown in a complete nutrient solution containing nitrogen as 1 mM ammonium nitrate. 4 days before the experiment, plants were transferred to N-free medium. For the assay, 100 μM ^15^[N]-urea was supplied to the medium for 15 min. Data are mean ±SD of three independent experiments and different letters above the bars indicate statistically significant differences (ANOVA, Student-Newman-Keuls*, P < 0.05, n*=3).

Phenotyping results were validated by ^15^[N]-urea influx assay using 6-weeks-old *Arabidopsis* plants. Col-0, *atdur3-3* and *atdur3-3* + ZmDUR3-A, −B, −C overexpression lines were grown in hydroponic culture in a complete nutrient solution containing 1 mM ammonium nitrate for 38 days before being transferred for 4 days in a N-free nutrient solution. At the time of the experiment, no phenotypical differences in root architectures were visible between different *Arabidopsis* lines under these growth conditions. When 100 μM ^15^[N]-urea was supplied to roots, all three ZmDUR3-overexpressing lines were able to take up urea, restoring the wild-type transport rates (Figure 8). In particular, the highest urea uptake rates were found in line B of the *atdur3-3* + ZmDUR3 overexpression line, while line -A and -C showed levels of urea uptake comparable to those in wild type plants.

## Discussion

Although urea is the most used N fertilizer worldwide, little is known on the capacity of crop plants to use urea *per se* as an N source. Maize is one of the crops supplied with huge amount of urea fertilizers and it is known that urea sustains N nutrition. However, it is not clear how much urea is directly taken up [23]. Therefore in this work, the high affinity urea uptake by maize roots was characterized and a high affinity urea transporter (ZmDUR3) identified and functionally characterized.

Among higher plants, the kinetic characterization of urea uptake was previously described only in *Arabidopsis* and rice [18,19]. In the present work, intact maize roots exposed to urea up to 300 μM, showed saturable kinetics of urea transport fitting into the Michaelis-Menten model (Figure 1). This behaviour is compatible with the presence of a high-affinity transport system for urea in maize roots, with kinetic features similar to those already characterized in other higher plants [18,19].

The kinetic assay in maize roots revealed an important aspect of urea uptake that has not been previously described in higher plants. Data showed that when maize plants were supplied with 1 mM urea for 4 hours, the affinity and capacity to take up this N source in the high-affinity concentration range (2.5-300 μM) increased in comparison to plants without urea pre-treatment (Figure 1). Thus, urea pre-treatment increases its own uptake, causing a modification of the kinetic parameters, which is very similar to the well-described physiological induction by substrate of the inducible high-affinity-nitrate transport system (iHATS) [24].

On the other hand, concerning the low-affinity transport system, the up-regulation of urea uptake by pre-treatment with urea was previously reported in *Arabidopsis* [25]. Results were inferred from influx assays performed by exposing plants to a high concentration of urea, 10 mM ^15^ N-urea (corresponding to 20 mM total N). The influx capacity of urea-fed plants (>300 μmol urea g^−1^ DW h^−1^) was higher than in N-starved plants or plants fed with ammonium nitrate or ammonium nitrate plus urea, which showed values around 200 μmol urea g^−1^ DW h^−1^. Thus, these data suggest that in *Arabidopsis* [25] and maize (Figure 1), roots are able to induce urea uptake when urea is available in the external medium. Moreover, as observed in the present work, the induction of HATS in maize roots might reflect an efficient response of plants by increasing the capacity of urea acquisition especially when this N source occurs at micromolar levels in the soil solution. Although after 24 hours high amount of external urea are taken up by the roots, the total concentration of urea as an intact molecule within maize plants did not increase (Additional file 1: Figure S1). So, the urea treatment seemed to have no effect on urea content in maize, similar results were also reported by Mérigout *et al.* [23]. This result may be explained by the high activity of the cytosolic urease enzyme, ubiquitously present in plant tissues, which has been shown to efficiently hydrolyse urea within the plant tissues [26]. Nevertheless, data here presented showed a transient modulation of urea content within the tissues suggesting a translocation of urea from roots to shoots.

Among higher plants, urea transporters have been identified only as orthologs of ScDUR3, an urea transporter of *S. cerevisiae*. Up to date, only AtDUR3 and OsDUR3, of *Arabidopsis* and rice, respectively have been functionally characterized, while in other monocots and dicots putative DUR3-orthologs were predicted by bioinformatics (Figure 2). In *Arabidopsis*, AtDUR3 has been described to be a major component of the high-affinity transport system, suggesting that also in other plants, the DUR3-orthologs might play a crucial role in urea acquisition. The expression level of DUR3 orthologs has been shown to be increased by the nitrogen deficiency in *Arabidopsis* and rice plants [18,19]. As reported for the orthologous gene in rice [19], the expression level of *ZmDUR3* coding for the putative urea transporter in maize is different among the tissues (Figure 3). The higher expression of the gene coding for DUR3 in the radical tissue might reflect its involvement in the mechanisms of urea acquisition from the root external medium. Roots of N-deficient plants treated with nitrogen sources exhibits divergent expression level of *DUR3* orthologs: in rice, *OsDUR3* is weakly induced after 3 hours of treatments with 1 mM urea [19], in *Arabidopsis*, 1 mM urea represses *AtDUR3* expression at 3 and 6 hours and induced it at 9 and 24 hours [18]. In maize plants, during the timespan when 1 mM urea induced an increase in the root capacity to take up urea, the expression level was decreasing (Figure 3) similarly to the variations found by Kojima *et al.* [18]. Therefore in the short term, the modulation in the root capacity to take up urea is not related to changes in the expression level of the gene *ZmDUR3*, suggesting the involvement of regulation mechanisms that do not operate at transcriptional level. Expression of *ZmDUR3* in a *dur3-S. cerevisiae* mutant demonstrated a functional urea transport (Figure 4). As *ZmDUR3*-transformants grew very slowly, a ZmDUR3-ORF was prepared with a lower GC content and therefore an optimized codon usage for *S. cerevisiae*. Therefore in the first part (10%) of the ORF, G and C in the third codon position were replaced with A or T generating codons which are more frequently used in yeast. Interestingly the *ZmDUR3*_*mod*_-transformants grew slightly faster than yeast mutants transformed with the unmodified *ZmDUR3*-ORF (Figure 4). Since the two constructs differed only at nucleotide level, the slow growth rate of *ZmDUR3*-ORF-expressing cells might be the consequence of a lower accumulation of ZmDUR3 protein possibly deriving from a lower transcription/translation of the native maize transgene in comparison to the *ZmDUR3*_*mod*_-transformed yeast.

These results highlight that especially for plant species with a high GC content, the ORF-optimization strategy may be a valid method to improve the expression of transgenes in heterologous systems like yeast or also in other model organisms allowing an easier molecular characterization of plant proteins.

The yeast complementation assay demonstrated that ZmDUR3 can mediate urea uptake from the external medium into the cells. With the aim to clarify the subcellular localization of ZmDUR3, tobacco protoplasts were transiently transformed with *ZmDUR3*_*mod*_-ORF fused with *GFP*. Results showed that the fluorescent signal was mostly detected in internal membranes (Figure 5), although the localization of a minor fraction of ZmDUR3-GFP on plasma membrane would be compatible with the observed signal. These localization results are comparable to those previously reported in *Arabidopsis* protoplasts for the orthologs of rice and *Arabidopsis*, OsDUR3 and AtDUR3 [19]. For these proteins, the fluorescent signals were not uniformly distributed at the periphery of protoplasts, indicating that the protein might be localized not only at the plasma membrane, but also in internal membranes.

Besides GFP-localization, further experimental evidences suggested that DUR3 might not exclusively be targeted to the plasma membrane. In particular, for AtDUR3 the plasma membrane localization in *Arabidopsis* root cells was previously described by two immunological approaches. Kojima *et al.* [18] used polyclonal antibodies against AtDUR3 in two independent analyses: a protein gel-blot analysis of membrane-protein fraction from *Arabidopsis* roots and an immunohistochemical assay on whole-mount root samples. Both immunological techniques gave the same results: although AtDUR3 localized at the plasma membrane, a fraction of the protein appeared to be localized in the cytoplasm. The authors suggested that a fraction of AtDUR3 might reside in endomembrane compartments, reflecting proteins that were moving to or from the plasma membrane [18].

Interestingly, in root cells, the subcellular-localization of another high affinity transporter (*Arabidopsis* Iron-Regulated Transporter 1, IRT1) was found to be mainly localized in the early endosomes [27] while at the plasma membrane the abundance of IRT1 was low and tightly regulated by an ubiquitin-dependent trafficking and turnover. The turnover of the IRT1 protein was investigated and the localization of IRT1 was explained by the authors as a result of a “rapid endocytosis and slower recycling to the plasma membrane, where it likely performs iron uptake from the soil, and is addressed to the lytic vacuole for turnover” [27]. The authors concluded that the internal traffic controls the amounts of IRT1 protein at the plasma membrane and therefore participates in the tight regulation of the nutrient uptake. These considerations about IRT1 suggest that the presence of ZmDUR3 in internal membranes may reflect a similar situation where the abundance of the protein at the plasma membrane is under control of a trafficking/recycling pathway. This hypothesis is further supported by the fact that the higher root uptake capacity of urea (Figure 1) was not accompanied by an overexpression of *ZmDUR3* (Figure 3).

To provide more detailed assessment of the molecular and physiological role of this maize transporter *in planta*, the overexpression of *ZmDUR3*_*mod*_ in a *dur3* mutant line of *Arabidopsis* was performed. All three overexpression lines were able to phenotypically recover the *dur3-*mutant (Figure 6a) and produced significantly higher plant biomass and root proliferation than *dur3* mutant and wild type (Figure 6a,b; Figure 7). This result might reflect a possible overexpression of the transgene in all the tissues of lines A, B and C, determining an improvement on the utilization of urea (translocation, allocation, redistribution) within the plants.

In short term 100 μM ^15^[N]-urea influx experiment (Figure 8), all three lines complement the mutant phenotype, reaching the highest uptake rates in line B. The differences in the uptake rates might be due to a different expression level of the transgene *ZmDUR3* in the three independent lines.

Moreover the influx experiment was performed at a micromolar concentration suggesting the capacity of ZmDUR3 to operate in the high affinity range. In conclusion, these evidences demonstrated the complementation of the mutant phenotype by ZmDUR3 and confirmed the physiological role of this protein as a high-affinity transporter of urea from soil into plants.

## Conclusions

For the first time, we report a physiological characterization of urea uptake in roots of intact maize plants. Results indicated that at micromolar urea concentrations (up to 300 μM urea), maize roots are able to take up this N source using a high affinity transport system characterized by saturable kinetics. Moreover, the pre-treatment of plants with urea increases their capacity to take up urea, showing that high-affinity uptake of urea is inducible by the substrate.

The capability of the identified ZmDUR3 to phenotypically complement *dur3* yeast and *Arabidopsis* mutants further demonstrates that *ZmDUR3* encodes a high-affinity urea uptake system in maize.

## Methods

### Maize growth conditions

Maize seeds (*Zea mays* L., cv. PR33T56, Pioneer Hi-bred Italia S.p.A., Parma, Italy) were germinated on a plastic net placed at the surface of an aerated 0.5 mM CaSO_4_ solution in a growth chamber at 25°C in the dark. After 3 days, the seedlings were transferred into an aerated hydroponic system containing 0.5 mM CaSO_4_ under controlled climatic conditions: day/night photoperiod, 16/8 h; light intensity, 220 μmol m^−2^ s^−1^; temperature (day/night) 25/20°C; relative humidity 70 to 80%. After 2 days (5-days-old) plants were transferred for a maximum of 24 h in a N-free nutrient solution containing (μM): KCl 5; CaSO_4_ 500; MgSO_4_ 100; KH_2_PO_4_ 175; NaFe-EDTA 20; H_3_BO_3_ 2.5; MnSO_4_ 0.2; ZnSO_4_ 0.2; CuSO_4_ 0.05; Na_2_MoO_4_ 0.05. N was supplied in the form of 1 mM CO (NH_2_)_2_ (urea-treated plants); or as control, plants were exposed to a N-free nutrient solution (control-plants). The pH of solution was adjusted to pH 6.0 with potassium hydroxide (KOH).

For the experiments of ^15^[N]-urea acquisition, urea-treated plants were exposed to nutrient solution containing 1 mM ^15^[N]-urea (98 atom% ^15^[N]; ISOTEC® Stable Isotopes, Sigma Aldrich, Milano, Italy).

### Measurement of net high-affinity urea uptake in maize plants

After 4 hours from the beginning of the N-treatment, roots of intact seedlings were immersed for 10 min, a time span during which uptake remained linear, in 40 ml of a constantly stirred and aerated solution containing 500 μM CaSO_4_ and up to 300 μM urea (2.5, 5, 10, 25, 50, 100, 200 or 300 μM urea). For each urea concentration, the uptake rates were determined using six urea-treated and six control-plants. Net uptake rate was measured as urea depletion from the solution per unit of time. Thus, samples of the solution (60 μl) were taken every 2 min and the urea content was determined by diacetylmonoxime and thiosemicarbazide colorimetric assay (modified from Killingsbaeck [28]). Therefore a 60 μl aliquot was mixed thoroughly with 120 μl of colour development reagent, which consisted of 1:1 mixed colour reagent [7% (v/v) 0.2 M diacetylmonoxime; 7% (v/v) 0.05 M thiosemicarbazide]: mixed acid reagent [20% (v/v) sulphuric acid (H_2_SO_4_); 0.06% (v/v) 74 mM ferric chloride hexahydrate in 9% (v/v) ortho-phosphoric acid]. The samples were incubated for 15 min at 99°C (lid temperature: 105°C) in a thermocycler. The samples were cooled 5 min on ice and the urea concentration was determined spectrophotometrically by measuring the absorbance at 540 nm using a microtiter plate reader. The uptake rates were expressed as μmol urea g^−1^ root FW h^−1^.

Kinetic parameters of the high-affinity urea uptake system (Vmax and Km) were calculated in the 2.5-300 μM concentration range by NonLinear Regression-Global Curve Fitting and the statistical analysis was performed by Normality Test (Shapiro-Wilk) using SigmaPlot 12.0 (Systat software, Point Richmond, USA).

### Determination of urea concentration

Root and leaf urea concentrations were measured in time-course (up to 24 hours of treatment) by colorimetric assay as described above (modified from Killingsbaeck [28]). Approximately 100 mg (fresh weight) of freeze plant tissues were milled and suspended in 1 ml of water at 99°C for 3 min. After centrifugation at 15000 g for 2 min, 60 μl of supernatant were incubated with 120 μl of colour-development reagent as previously described. Kojima *et al.* [18] reported that ureides allantoin, ornithine, arginine and uric acid did not interfere with the urea determination by diacetylmonoxime and thiosemicarbazide.

### ^15^[N]-analysis

Approximately 1 mg of dried root and leaf tissues was transferred into a tin capsule for measurement of δ^15^N in one run. The analysis was carried out using a Delta V isotope ratio mass spectrometer (Thermo Scientific, Bremen, Germany) equipped with a Flash EA 1112 Elemental Analyser (Thermo Scientific, Bremen, Germany). The isotope ratios were expressed in δ ‰ versus air for δ^15^N according to the following formula: δ ‰ = [(R_sample_–R_standard_)/R_standard_]⋅ 1000 where R_sample_ is the isotope ratio measured for the sample and R_standard_ is the isotope ratio of the international standard. R is the abundance ratio of the minor, heavier isotope of the element to the major, lighter isotope, as ^15^ N/^14^ N. The isotope values were calculated against international reference materials: L-glutamic acid USGS 41, ammonium sulphate IAEA-N-2 (IAEA-International Atomic Energy Agency, Vienna, Austria) and urea 33802174IVA (IVA Analysentechnik e.k.). The uncertainty of the nitrogen isotopic determination was ± 0.3‰.

### Molecular work

#### RNA extraction

Total RNA was isolated from roots and leaves of maize plants. The RNA extractions were performed using the Invisorb Spin Plant RNA kit (Stratec Molecular, Berlin, Germany) as reported in the manufacturer’s instructions (http://www.invitek.de/). The integrity of RNA was qualitatively checked on a 1% agarose gel and quantified by spectrophotometer Nanodrop 2000 instrument (Thermo Scientific, Wilmington, USA).

#### Real-time RT-PCR experiments

One μg of total RNA was retrotranscribed in cDNA using Oligo-dT_23_ and the Superscript II Reverse Transcriptase (Gibco BRL, Basel, Switzerland), a RNase H derivative of moloney murine leukemia virus, according to the manufacturer’s protocol. After RNA digestion with 1 U RNase A (USB, Cleveland, USA) for 1 h at 37°C, gene expression analyses were performed by adding 0.16 μl of the cDNA to the real-time RT-PCR complete mix, FluoCycle™ sybr green (20 μl final volume; Euroclone, Pero, Italy), in a DNA Engine Opticon Real Time PCR Detection (Biorad, Hercules, USA).

Based on a *ZmDUR3-*EST sequence (BQ164112), specific primers (Tm = 58°C) were designed to generate 109 bp PCR product: CCTCAATCTGGTGGGTGTCT and ATTGGCCTTTCTCCACAGC (PCR efficiency 81%). Real-time RT-PCR analyses were performed in triplicates on three independent experiments. The analyses of real-time result were performed using Opticon Monitor 2 software (Biorad) and R (version 2.9.0; http://www.r-project.org/) with the qPCR package (version 1.1-8; [29]). Efficiencies of amplification were calculated following the authors’ indications [29]. Data were normalized with respect to the transcript level of the housekeeping gene (*ZmRPS4,* AF013487, GCAACGTTGTCATGGTGACT and CTCCACGTGAATGGTCTCAA, PCR efficiency 86%) using the 2^-ΔΔ*C*T^ method, where *ΔΔC*_T_ = (C_T,Target_ − C_T,HK_)_Time x_ − (C_T,Target_ − C_T,HK_)_Time 0_ [30].

#### ZmDUR3-ORF cloning

In order to clone *ZmDUR3*-ORF, two reverse transcription reactions (RT-reaction) were performed, one reaction was transcribed using Oligo-dT_23_ while in the other reaction a specific primer for the *ZmDUR3*-ORF was used (2 μM; reverse 5′-CAGGAATGAGGTGAAGAGCGCGAAGAAGGCGC-3′). For each reaction, 2 μg of total RNA were reverse transcribed.

Since the first 200 bp of the predicted ORF sequence were high in GC%, the *ZmDUR3*-ORF was amplified in two separate PCR-reactions; *i.e.* generating two fragments with an overlap of 20 bp, which were subsequently assembled using Assembly-PCR. The 5′-fragment (192 bp) covered the first part of the ORF sequence (from +1 to +192 bp) and was amplified from cDNA obtained with the ZmDUR3-specific primer (50 ng as template of PCR reaction). The 3′-fragment (2024 bp) covered most of the remaining ORF sequence (from +172 up to +2196) and was amplified using cDNA obtained with oligo-dT_23_ (100 ng as template of PCR reaction).

All PCR reactions were performed in a 50 μL reaction volume containing 5 × GC Buffer for Phusion® High-Fidelity DNA Polymerase, 0.2 mM ATP, 0.2 mM TTP, 0.3 mM GTP, 0.3 mM CTP, 0.4 μM forward primer, 0.4 μM reverse primer, 2 U Phusion® High-Fidelity DNA Polymerase (New England Biolabs (UK) Ltd., Hitchin, United Kingdom) following the temperature protocol: 98°C for 30 s; 98°C for 10 s, 58 - 68°C for 30 s, 72°C for 30 s to 2 min, 35 cycles; 72°C for 10 min. The 5′-fragment was amplified using 5′-CGGAATTCATGGCCGCTGGCGGCGCCGGC-3′ as forward primer and 5′-CAGGAATGAGGTGAAGAGCGCGAAGAAGGCGC-3′ as reverse primer (Tm = 68°C, elongation at 72°C for 30 s). The 3′-fragment was amplified using 5′-TTCTTCGCGCTCTTCACCTC-3′ as forward primer and 5′-CGCGGATCCTTAAGCTAGCGAAAGATTATCTTCATC-3′ as reverse primer (Tm = 58°C, elongation at 72°C for 2 min). The 5′- and 3′-fragments of the *ZmDUR3*-ORF were assembled using the approach of Assembly PCR. The PCR reaction was carried out with 10 ng 5′-fragment and 10 ng 3′-fragment*,* as template; using 5′-CGGAATTCATGGCCGCTGGCGGCGCCGGC-3′ as forward primer and 5′-CGCGGATCCTTAAGCTAGCGAAAGATTATCTTCATC-as reverse primer (Tm = 62°C, elongation at 72°C for 1 min 30 s). The full-length *ZmDUR3*-ORF [GenBank: KJ652242] was amplified and cloned into the *S. cerevisiae* expression vector pDR197 [21] using the restriction sites for *EcoRI* and *BamHI*. The nucleotide sequence was verified by sequencing.

#### ZmDUR3_mod_-ORF *cloning*

In order to reduce the GC content and to facilitate the expression of *ZmDUR3* in heterologous organisms, 48 nucleotides in the first 216 nt of *ZmDUR3* were modified. These modifications are all synonymous substitutions occurring only at the third base of the codons (the codon-usage preference in yeast was chosen as described by http://www.kazusa.or.jp/codon/). This modified *ZmDUR3,* called *ZmDUR3*_*mod*_ [GenBank: KJ652243], differs from the *ZmDUR3* only at nucleotide level, while the encoded amino acids remain unchanged (Additional file 5: Figure S5).

The modified region was obtained by assembling two primers, Assembly-1 Primer *(*5′-GGAATTC**ATG**GCTGCTGGTGGTGCTGGTGCTTGTCCTCCACCAGGTCTAGGTTTTGGTGGTGAATATTATTCTGTTGTTGAT*GGTGCTTGTAGTCGTGATGG* -3′) and Assembly-2 Primer (5′-*GGTGCTTGTAGTCGTGATGG*TAGCTTTTTTGGCGGTAAACCAGTTCTAGCTCAAGCTGTTGGTTATGCTGTCGTTCTTGGTTTTGGTGCT*TTCTTCGCGCTCTTCACCTC*-3′), which were synthetized *in vitro* (Microsynth AG, Balgach, Switzerland).

Two consecutive Assembly PCR reactions were performed to add the long primers to the 3′-fragment.

In the first PCR reaction, 10 ng of 3′-fragment were used as template, while Assembly-2 Primer and 3′-fragment were assembled by PCR, *i.e.* 10 ng of 3′-fragment were used as template; while Assembly-2 Primer and 5′-CGCGGATCCTTAAGCTAGCGAAAGATTATCTTCATC-3′ were used as forward and reverse primers, respectively (Tm = 62°C elongation at 72°C for 1 min 30 s). 10 ng of purified PCR product were used as template for the consecutive PCR with forward and reverse primers: Assembly-1 Primer and 5′-CGCGGATCCTTAAGCTAGCGAAAGATTATCTTCATC-3′ (Tm = 62°C, elongation at 72°C for 1 min 30 s).

Using the restriction sites *EcoRI* and *BamHI*, the full-length *ZmDUR3*_*mod*_-ORF was cloned into vector pDR197 [21] and sequenced.

Although the optimization of codon usage in ZmDUR3mod was developed for a better expression in yeast, the modified sequence was also used to perform the functional characterization of DUR3 in tobacco protoplasts and *A. thaliana,* since also in these latter organisms a high GC content might interfere with the translation of the transcripts.

### Expression in *Saccharomyces cerevisiae*

*S. cerevisiae* strain YNVWI (Δura3, Δdur3 [13]) was transformed with vector pDR197 (negative control) or plasmids harbouring the ORF sequences (pDR197-*ZmDUR3* and pDR197-*ZmDUR3*_*mod*_) as described by Liu *et al.* [13]. Transformants were first selected on synthetic dextrose minimal medium [31] with Oxoid agar (Difco, Detroit, USA) [32]. Single colonies were tested on urea (1, 2 or 3 mM) or ammonium sulphate (0.5% w/v) as sole N source. The pH of the medium was adjusted with 1 M KOH (pH 5.6). The cells were grown for 2–3 days at 28°C.

### Protein localization in *Nicotiana tabacum* protoplasts

For transient expression of *ZmDUR3*_*mod*_ in tobacco protoplasts, two plasmids harbouring the sequence for the Green Fluorescent Protein (GFP) were fused at the N- or C-terminus of ZmDUR3 using vectors pUC18-Sp-GFP6 and pUC18-GFP5T-Sp [22]. *ZmDUR3*_*mod*_-ORF sequence without stop codon was amplified using primers (5′-ATAACTAGT**ATG**GCTGCTGGTGGTGCTGG-3′, 5′-ATAtAGATCTGCAGCTAGCGAAAGATTATCTTCATCG-3′), and cloned into pUC18-Sp-GFP6 using the *SpeI* and *BglII* sites, yielding *ZmDUR3*_*mod*_*: GFP*. On the other hand, to obtain the *GFP: ZmDUR3*_*mod*_ construct, the *ZmDUR3*_*mod*_-ORF sequence with stop codon was amplified using primers (5′-ATATCTAGA**ATG**GCTGCTGGTGGTGCTGG-3′, 5′-ATAATGCATTTAAGCTAGCGAAAGATTATCTTCATCG-3′), and cloned into pUC18-GFP5T-Sp using the *NheI* and *PstI* sites.

Protoplast isolation and transformation was performed as described earlier [33]. For co-localization experiments pUC-PTR1-Sp-EYFP [22] was used as marker for the plasma membrane. Tobacco protoplasts were co-transformed with either pUC18-*ZmDUR3*_*mod*_-GFP6 or pUC18-GFP5T-*ZmDUR3*_*mod*_ and pUC-PTR1-Sp-EYFP. As control, free GFP (pUC18-GFP5T-Sp) was transiently expressed in tobacco protoplasts. As reported by Komarova *et al.* [22], protoplasts were examined with a SP2 AOBS confocal microscope (Leica Microsystems, Wetzlar, Germany), excited with an argon laser at 458 nm for GFP and 514 nm for YFP. Fluorescence was detected at 492–511 nm for GFP, at 545–590 nm for YFP and 628–768 nm for chlorophyll epifluorescence detection. Diameter of tobacco protoplasts was approximately 40 μm.

### Generation of *ZmDUR3*_*mod*_*-*overexpressing *Arabidopsis* lines and growth phenotyping

The *ZmDUR3*_*mod*_-ORF was excised from pDR197-*ZmDUR3*_*mod*_ using *EcoRI* and *BamHI* and ligated into vector pBF1 [34] at the *EcoRI* and *BglII* sites. Using this pBF1- *ZmDUR3*_*mod*_ construct as template, the *ZmDUR3*_*mod*_-ORF was amplified using primers (5′-ATTTAGGTGACACTATAG-3′, 5′-CGCGGATCCTTAAGCTAGCGAAAGATTATCTTCATC -3′) and cloned into the final vector pCHF5 [35] in the *BamHI* site, generating a construct named pCHF5-*ZmDUR3*_*mod*_. *Arabidopsis atdur3-3* plants [18] were transformed by dipping inflorescences into a cell suspension (OD600 = 0.6) of *Agrobacterium tumefaciens* GV3101 harbouring pCHF5-*ZmDUR3*_*mod*_, as described by Clough & Bent [36]. Harvested seeds were germinated on soil; plants at two-leaf-stage were treated with glufosinate (150 mg l^−1^; BASTA® 200, Bayer CropScience Deutschland GmbH, Langenfeld, Germany) to select transformed lines. The experiments were performed using independent *ZmDUR3*-overexpressing lines of T2 or T3 generation.

For growth complementation tests, surface-sterilized seeds were grown on agar plates as described by Kojima *et al.* [18]. Plants were grown on modified half-strength Murashige and Skoog (MS) medium without N, supplemented with 1 μM NiCl_2_ and 50 μM KNO_3_. Either 500 μM NH_4_NO_3_ or 500, 1000 and 3000 μM urea were added as N sources, alternatively no N was added (negative control). Col-0, *atdur3-3* and three *atdur3-3* transformed lines (*atdur3-3* + ZmDUR3-A, −B, −C overexpression lines) were cultured for 16 days in a growth chamber with photoperiod, 24 h; light intensity, 220 μmol m^−2^ s^−1^; temperature, 20-22°C; relative humidity, 70 to 80%.

### Hydroponic culture of *Arabidopsis* plants and ^15^[N]-urea root uptake

*Arabidopsis thaliana* seeds (Col-0; *atdur3-3*; *atdur3-3* + ZmDUR3-A, −B, −C overexpression lines) were germinated on half strength MS-agar medium as described by Norén *et al.* [37]. After 10 days, the seedlings were transferred for 6 weeks to hydroponic conditions as previously described by Kojima *et al.* [18]. During the entire growth period N was supplied as 1 mM NH_4_NO_3_. 4 days before the experiment, plants were transferred to medium lacking N (no N).

Urea influx measurements into plant roots were conducted after rinsing the roots in 0.5 mM CaSO_4_ solution for 1 min, followed by incubation for 15 min in nutrient solution containing 100 μM of ^15^[N]-urea (98 atom% ^15^ N; ISOTEC® Stable Isotopes, Sigma Aldrich, Milano, Italy) as the sole N source. After a final rinse of 1 min in 10 mM non-labelled, ice-cold urea and a second rinse of 1 min in 0.5 mM CaSO_4_ solution, the *Arabidopsis* roots were sampled and dried at 40°C and analysed as previously described.

### Phylogenetic and statistical analyses

Phylogenetic analyses were conducted using MEGA version 6 software [38]. The tree was constructed by aligning the protein sequences by Clustal-W and the evolutionary history was inferred using the Neighbor-Joining method. The percentage of replicate trees in which the associated taxa clustered together in the bootstrap test (1000 replicates) are shown in Figure 2 next to the branches. The tree is drawn to scale, with branch lengths in the same units as those of the evolutionary distances used to infer the phylogenetic tree. The evolutionary distances were computed using the Poisson correction method and are in the units of the number of amino acid substitutions per site.

For the experiments with maize and *Arabidopsis* plants, three independent experiments were performed using six (if not otherwise specified) plants for each sample; each sample was measured performing three technical replicates. Statistical significance was determined by one-way analysis of variances (ANOVA) using Student-Newman-Keuls test, taking *P* < 0.05 as significant. Statistical analysis were performed using SigmaPlot Version 12.0 software.

## Additional files

Additional file 1: Figure S1. Urea concentration in roots and shoots of maize in response to the presence of urea in hydroponic solution. 5-day-old maize plants were exposed for a maximum of 24 h to a nutrient solution without any nitrogen source (*Control* plants) or supplied with 1 mM urea as a sole nitrogen source (*Urea* treated plants). Values are means ± SD of three independent experiments (ANOVA, Student-Newman-Keuls*, P < 0.05, n* = 3). *Capital letters* are referred to the statistical differences in the roots, while *lower letters* are referred to shoots. Additional file 2: Figure S2. Schematic representation of the position of exons of the predicted (a) and isolated (b) sequence of *ZmDUR3-*ORF on the genomic sequence (from +1 bp of start codon, to stop codon +5567 bp). In the table, the numbers are referred to the position on the genomic locus coding for ZmDUR3. (*) six nucleotides are not present in the fourth exon of the isolated *ZmDUR3*-ORF. Additional file 3: Figure S3. Amino-acid alignment of ZmDUR3, OsDUR3 and AtDUR3. The alignment was made using Clustal-W. Additional file 4: Figure S4. Comparison of predicted topologies of ZmDUR3 and OsDUR3 (prediction was performed by http://topcons.cbr.su.se/). Additional file 5: Figure S5. Nucleotide differences between *ZmDUR3* (upper row) and *ZmDUR3*_*mod*_ (lower row) sequences. To generate *ZmDUR3*_*mod*_ (KJ652243), the nucleotide sequence of the first 216 nucleotides of *ZmDUR3* (KJ652242) was modified by substituting only the third base of the codons (highlighted in yellow), with no difference occurring at the amino acid level.
