# Supplementary figures and images for: Isolation and functional characterization of a high affinity urea transporter from roots of Zea mays
Source: BMC Plant Biol. 2014 Aug 29;14:222. doi: 10.1186/s12870-014-0222-6 (PMC4160556; doi:10.1186/s12870-014-0222-6)

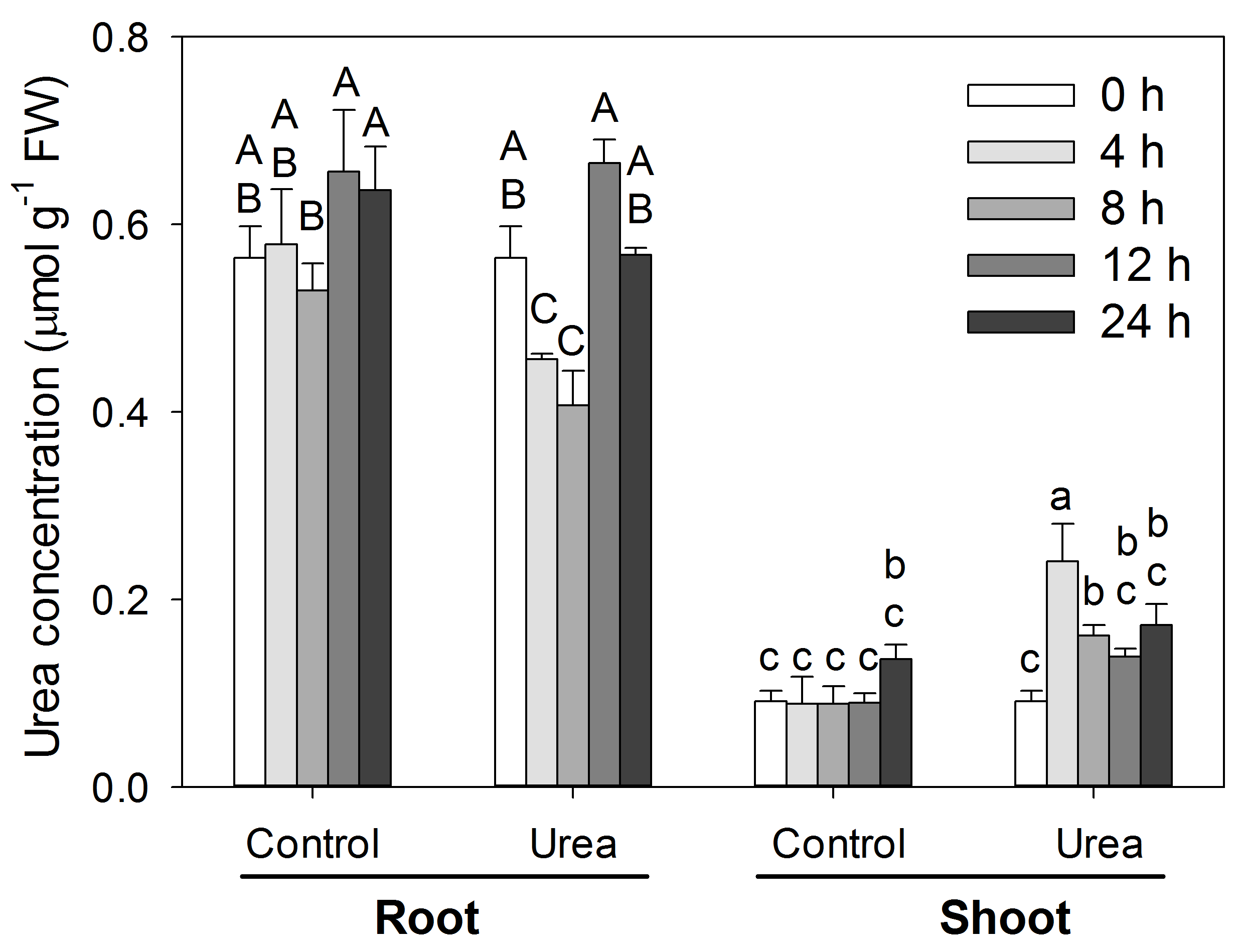

Supplement: Additional file 1: Figure S1. — Urea concentration in roots and shoots of maize in response to the presence of urea in hydroponic solution. 5-day-old maize plants were exposed for a maximum of 24 h to a nutrient solution without any nitrogen source (Control plants) or supplied with 1 mM urea as a sole nitrogen source (Urea treated plants). Values are means ± SD of three independent experiments (ANOVA, Student-Newman-Keuls, P < 0.05, n = 3). Capital letters are referred to the statistical differences in the roots, while lower letters are referred to shoots. [file 12870_2014_222_MOESM1_ESM.tiff]

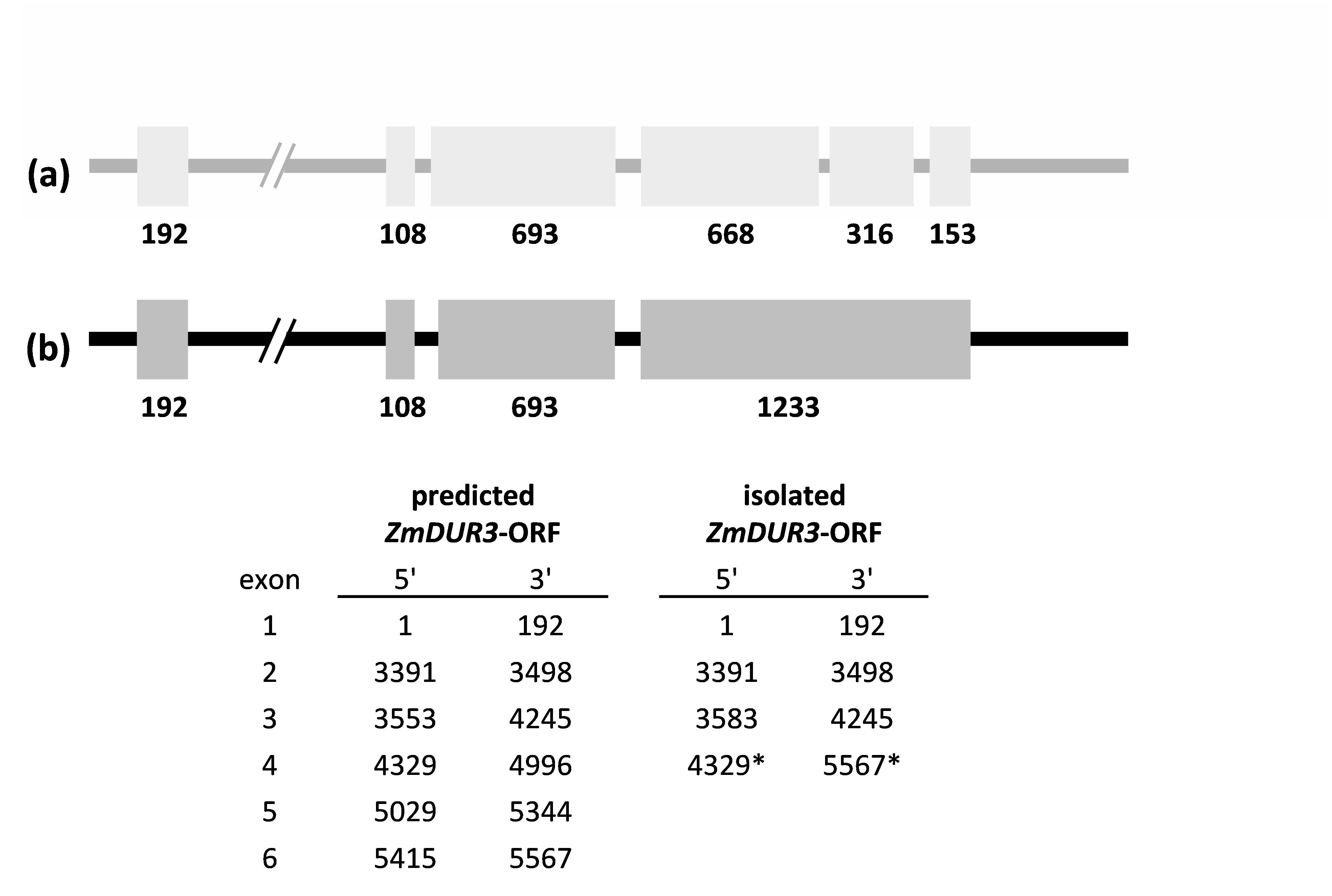

Supplement: Additional file 2: Figure S2. — Schematic representation of the position of exons of the predicted (a) and isolated (b) sequence of ZmDUR3-ORF on the genomic sequence (from +1 bp of start codon, to stop codon +5567 bp). In the table, the numbers are referred to the position on the genomic locus coding for ZmDUR3. (*) six nucleotides are not present in the fourth exon of the isolated ZmDUR3-ORF. [file 12870_2014_222_MOESM2_ESM.tiff]

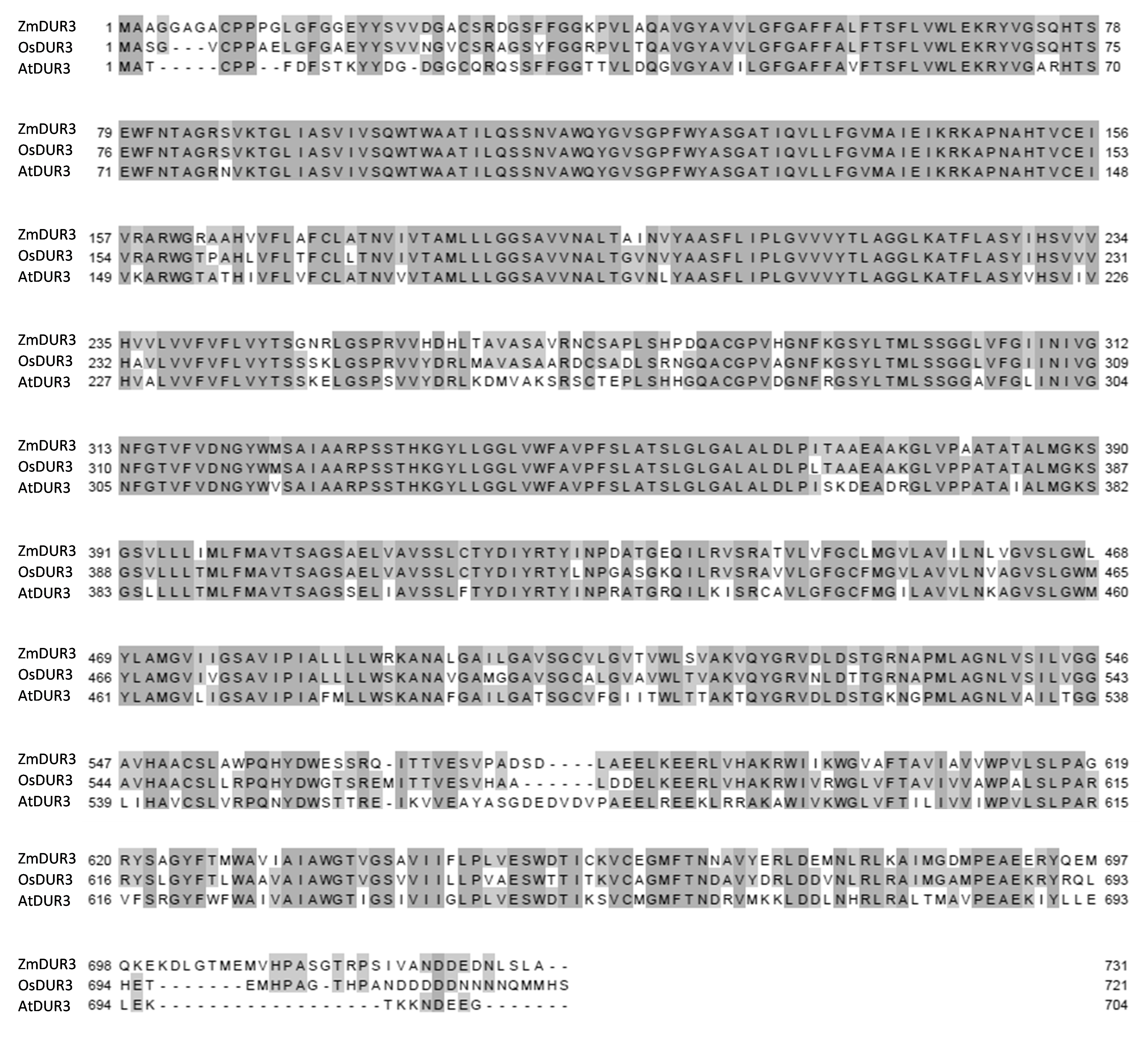

Supplement: Additional file 3: Figure S3. — Amino-acid alignment of ZmDUR3, OsDUR3 and AtDUR3. The alignment was made using Clustal-W. [file 12870_2014_222_MOESM3_ESM.tiff]

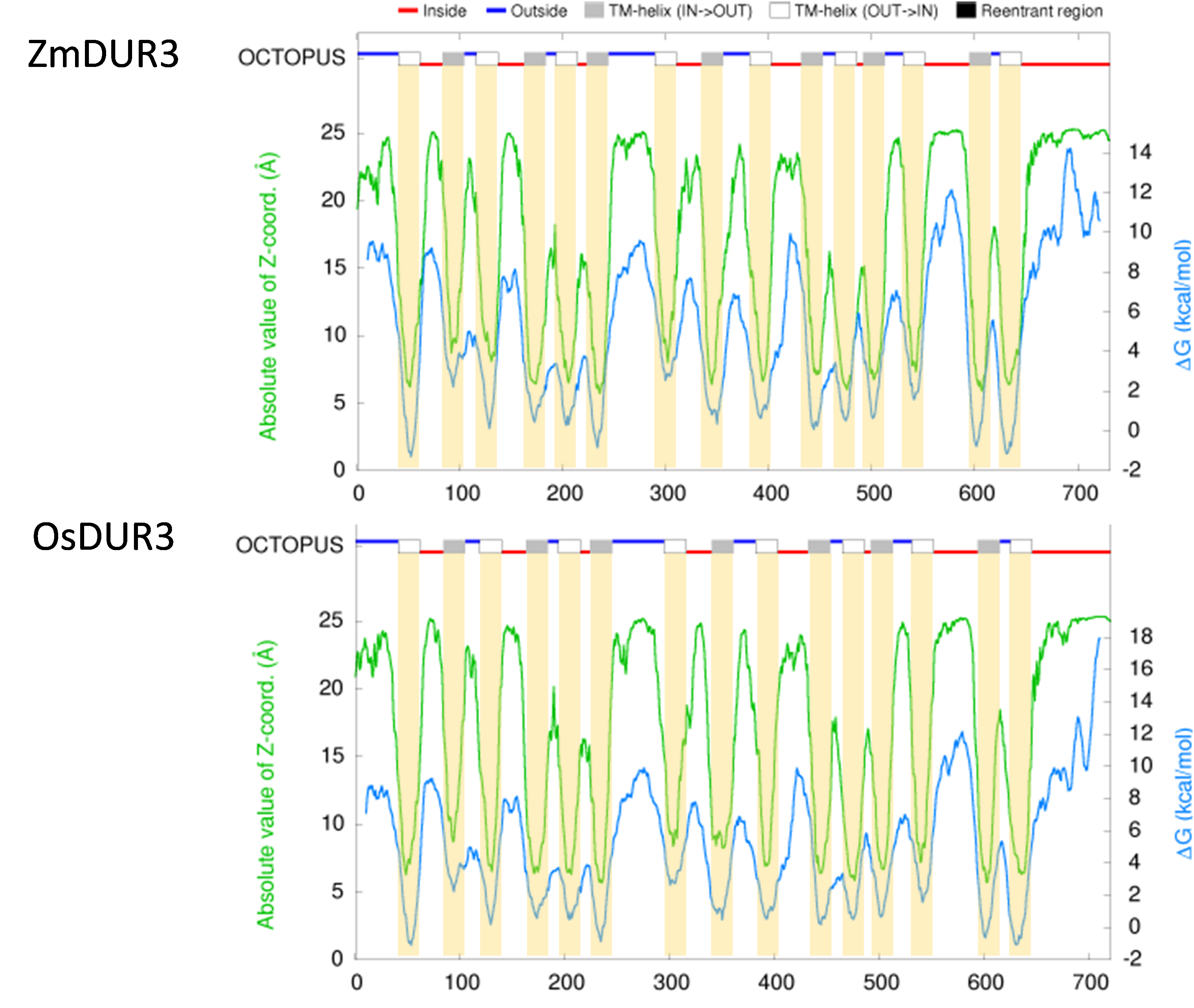

Supplement: Additional file 4: Figure S4. — Comparison of predicted topologies of ZmDUR3 and OsDUR3 (prediction was performed by http://topcons.cbr.su.se/). [file 12870_2014_222_MOESM4_ESM.tiff]

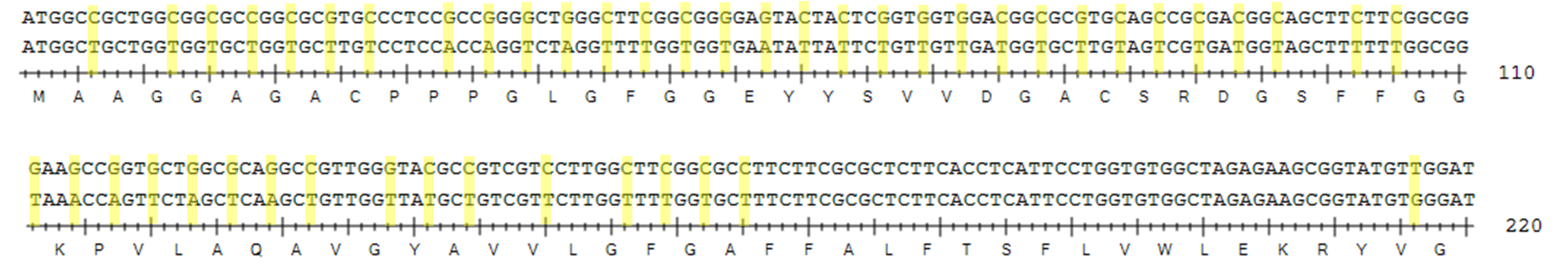

Supplement: Additional file 5: Figure S5. — Nucleotide differences between ZmDUR3 (upper row) and ZmDUR3 mod (lower row) sequences. To generate ZmDUR3 mod (KJ652243), the nucleotide sequence of the first 216 nucleotides of ZmDUR3 (KJ652242) was modified by substituting only the third base of the codons (highlighted in yellow), with no difference occurring at the amino acid level. [file 12870_2014_222_MOESM5_ESM.tiff]
